# Evaluation of anthropometric and virologic data in newborn from HIV positive mothers

**DOI:** 10.1186/1471-2334-14-S4-P10

**Published:** 2014-05-29

**Authors:** Simona Claudia Cambrea, Doina Eugenia Tănase, Maria Margareta Ilie, Dalia Sorina Carp, Elena Dumea, Stela Halichidis, Lucian Cristian Petcu

**Affiliations:** 1 Clinical Infectious Diseases Hospital of Constanța, Romania; 2 Faculty of Medicine, “Ovidius” University, Constanța, Romania

## 

Constanța used to be one of the most affected counties of Romania by HIV in children. Nowadays in Constanța there are an increasing number of HIV positive young women at fertile age who have babies. Even though there were implemented active measures for prevention of mother to child HIV transmission we still diagnose mothers with HIV after delivery. On the other hand HIV infection increases the risk of intrauterine growth restriction (IUGR) of newborn.

The objectives of this study were to evaluate the proportion of children born from HIV positive women who presented IUGR; and to evaluate materno-fetal transmission rate of HIV in Constanța County.

We performed a retrospective study on the relevant parameters in newborns and mothers: demographic data; CD4 count and HIV viral load in last trimester of pregnancy of mothers; HIV viral load in newborn. We analyzed anthropometric data of the newborn: weight, length, cranial circumference, and Apgar score. Statistical analysis was performed using SPSS version 19.

Over a period of 6 years and 2 months, 135 newborn from 117 HIV+ mothers have been monitored. From all 135 children born from HIV positive mothers 5 were HIV positive. The median age in mothers was 23 and mean 23.08 (range: 17 to 39, SD=3.58). The mean Apgar score in newborns was 8.47 (range: 2 to 10, SD=1.202), and median 9. The mean birth weight in newborns was 2692 g (range: 1000 to 3900, SD=516.389), and median 2700 g. The proportion of children with birth weight less than 10^th^ percentile was 58.05%. The mean length was 47.66 cm (range: 39 to 52, SD = 2.75), with a proportion of children below the 10^th^ percentile of 27.4%. Infants who presented below the 10^th^ percentile for weight and length were 23%. About 21.48% of infants were below the 10^th^ percentile for weight, length and cranial circumference. Mean CD4 count in mothers in third trimester of pregnancy was 415.14 (range 27-1156), and median 397. 53.3% of mothers were with HIV viral load undetectable in the last trimester of pregnancy. In the studied period the mortality rate was 6.7% in children and 5.9% in mothers.

The materno-fetal rate of HIV transmission was 3.7%. More than half (58.05%) of the infants born to HIV positive mothers were small for gestational age. 23% of infants were with IUGR and 23% of them presented symmetrical IUGR.

